# Campus Smoking Policies and Smoking-Related Twitter Posts Originating From California Public Universities: Retrospective Study

**DOI:** 10.2196/33331

**Published:** 2021-12-24

**Authors:** Joshua S Yang, Raphael E Cuomo, Vidya Purushothaman, Matthew Nali, Neal Shah, Cortni Bardier, Nick Obradovich, Tim Mackey

**Affiliations:** 1 Department of Public Health California State University Fullerton Fullerton, CA United States; 2 Global Health Policy and Data Institute San Diego, CA United States; 3 Department of Anesthesiology University of California San Diego San Diego, CA United States; 4 S-3 Research San Diego, CA United States; 5 Global Health Program Department of Anthropology University of California, San Diego La Jolla, CA United States; 6 Center for Humans and Machines Max Planck Institute for Human Development Berlin Germany

**Keywords:** tobacco-free policies, social media, colleges and universities, smoking, smoking, smoking policy, campus policy, tobacco use, Twitter analysis, smoke-free, tobacco-free, Twitter, college students, students, campus, health policy

## Abstract

**Background:**

The number of colleges and universities with smoke- or tobacco-free campus policies has been increasing. The effects of campus smoking policies on overall sentiment, particularly among young adult populations, are more difficult to assess owing to the changing tobacco and e-cigarette product landscape and differential attitudes toward policy implementation and enforcement.

**Objective:**

The goal of the study was to retrospectively assess the campus climate toward tobacco use by comparing tweets from California universities with and those without smoke- or tobacco-free campus policies.

**Methods:**

Geolocated Twitter posts from 2015 were collected using the Twitter public application programming interface in combination with cloud computing services on Amazon Web Services. Posts were filtered for tobacco products and behavior-related keywords. A total of 42,877,339 posts were collected from 2015, with 2837 originating from a University of California or California State University system campus, and 758 of these manually verified as being about smoking. Chi-square tests were conducted to determine if there were significant differences in tweet user sentiments between campuses that were smoke- or tobacco-free (all University of California campuses and California State University, Fullerton) compared to those that were not. A separate content analysis of tweets included in chi-square tests was conducted to identify major themes by campus smoking policy status.

**Results:**

The percentage of positive sentiment tweets toward tobacco use was higher on campuses without a smoke- or tobacco-free campus policy than on campuses with a smoke- or tobacco-free campus policy (76.7% vs 66.4%, *P*=.03). Higher positive sentiment on campuses without a smoke- or tobacco-free campus policy may have been driven by general comments about one’s own smoking behavior and comments about smoking as a general behavior. Positive sentiment tweets originating from campuses without a smoke- or tobacco-free policy had greater variation in tweet type, which may have also contributed to differences in sentiment among universities.

**Conclusions:**

Our study introduces preliminary data suggesting that campus smoke- and tobacco-free policies are associated with a reduction in positive sentiment toward smoking. However, continued expressions and intentions to smoke and reports of one’s own smoking among Twitter users suggest a need for more research to better understand the dynamics between implementation of smoke- and tobacco-free policies and resulting tobacco behavioral sentiment.

## Introduction

The number of colleges and universities with smoke- or tobacco-free campus policies has been increasing [1-4]. As of July 2020, there were an estimated 2542 completely smoke-free campus sites (including 2104 completely tobacco-free sites), 2176 of which prohibit e-cigarette use everywhere [5]. Existing evidence suggests that smoke- and tobacco-free campus policies are well-received by the campus community [6,7] and norms shift to greater disapproval of tobacco use on campus [7]. Smoking rates appear to decline after the implementation of smoke- and tobacco-free campus policies [8,9], though e-cigarette use may increase after smoking restrictions are implemented [9,10]. Comparison of policies across universities suggests that stronger policies are associated with reduced second-hand smoke exposure [11], smoking behavior [11,12], and seeing others smoking [12].

The effects of campus smoking policies on overall sentiment, particularly among young adults, are more difficult to assess given the changing tobacco and e-cigarette product landscape and differential attitudes toward policy implementation and enforcement. Geolocating social media posts to specific spatiotemporal areas allows for comparison of in situ smoking-related attitudes and behaviors, including between campuses with and without smoke- or tobacco-free campuses. Hence, geolocation information available from publicly available social media Twitter posts represents an opportunity to garner infoveillance-generated insights retrospective to the policy implementation periods.

Public 4-year universities in California provide a unique comparison for assessing tobacco-related attitudes and behaviors during policy implementation. The state has two public 4-year university systems, the University of California (UC) and California State University (CSU). As of January 2014, a systemwide tobacco-free policy went into effect on all UC campuses prohibiting tobacco use, including e-cigarettes, on campus grounds [13]. The CSU followed suit with a systemwide policy effective September 2017 [14]. The intervening period provided an opportunity to assess the impact of the different system policies on campus tobacco-related attitudes and behaviors. Hence, the goal of the study was to retrospectively compare campus climate toward tobacco use (without respect to type of smoking product) between universities with and those without smoke- or tobacco-free campus policies using geolocated data from Twitter.

## Methods

### Data Collection

Geolocated Twitter posts from 2015, a year after which the UC system had enacted its systemwide tobacco-free policy and prior to enactment in the CSU system, were used to conduct a retrospective analysis of smoking-themed tweets geofenced from UC and CSU campuses (one CSU campus—Fullerton—enacted its own smokefree policy effective August 2013). Data collection was conducted using the Twitter public application programming interface in combination with cloud computing services on Amazon Web Services. Data were filtered for messages that included geospatial coordinates enabled by users. These data were collected in JSON format and stored in a relational database, with information on the date and time of the post, hyperlink to the original tweet, text of the tweet, and geospatial coordinates.

Posts were selected for further analysis if they included any of the following keywords associated with tobacco products and behavior: *bidis*, *cigarette*, *cigarettes*, *cigarillos*, *cigars*, *ciggie*, *class*, *dip*, *e-cig*, *hookah*, *huqqa*, *joint*, *JUUL*, *kreteks*, *Marlboro*, *Newport*, *njoy*, *pipe*, *roll-up*, *shag*, *smoke*, *smoking*, *snuff*, *snus*, *tobacco*, *vape*, *vaped*, *vapejuice*, *vaper*, *vapes*, *vapine*, *vapor*, *waterpipe*, *waxpen*, or *weed*. Keywords were selected and adopted on the basis of prior studies [15,16] and because they were related to college life or tobacco products according to manual searches conducted on Twitter. For the sake of parsimony, the top 2 leading brands of cigarette sold in the United States (Marlboro and Newport), which were also the most commonly mentioned brands in tweets related to college life as revealed from manual searches conducted on Twitter, were also included in the list of keywords.

Tweets that included these keywords were then further filtered to identify those physically originating (ie, geofenced) from CSU or UC campuses using base maps of these schools available from the Stanford Prevention Research Center [17], which the authors visually assessed for concurrent validity with school boundaries in satellite imagery via Google Maps. Latitude and longitude coordinates for posts containing keywords were loaded into ArcGIS Desktop (version 10.6) and the clipping function was used to omit tweets outside of CSU or UC campus shape files.

### Quantitative Data Analysis

Human annotators manually assessed posts relating to the study theme corresponding to user-generated tobacco products or related behavior, for face validity. Human annotators were trained in tobacco research and have participated in prior research infoveillance research [18,19]. Posts were manually annotated for positive, negative, or neutral sentiment toward smoking. These attributes were selected by 2 authors, with high interrater reliability (Cohen κ=0.96). Discrepancies were resolved through discussion among all authors. Chi-square tests were conducted to determine if there were significant differences in sentiment between campuses that were smoke- or tobacco-free (all UC campuses and CSU, Fullerton) compared to those that were not.

### Qualitative Content Analysis

A separate content analysis of tweets with smoking-related sentiment was conducted by the first author to identify central themes [20]. Tweets were imported into Atlas.ti (version 8) [21]. Each tweet was a recording unit and mutually exclusive. All tweets were coded for the smoking policy of the campus from which it originated (tobacco- or smoke-free vs non–tobacco- or –smoke-free). A general inductive approach was then used to develop a coding framework for tweets to assess thematic content [22]. Once the coding scheme was developed, tweets were coded and analyzed for major themes by campus smoking policy type.

## Results

**Figure 1:**
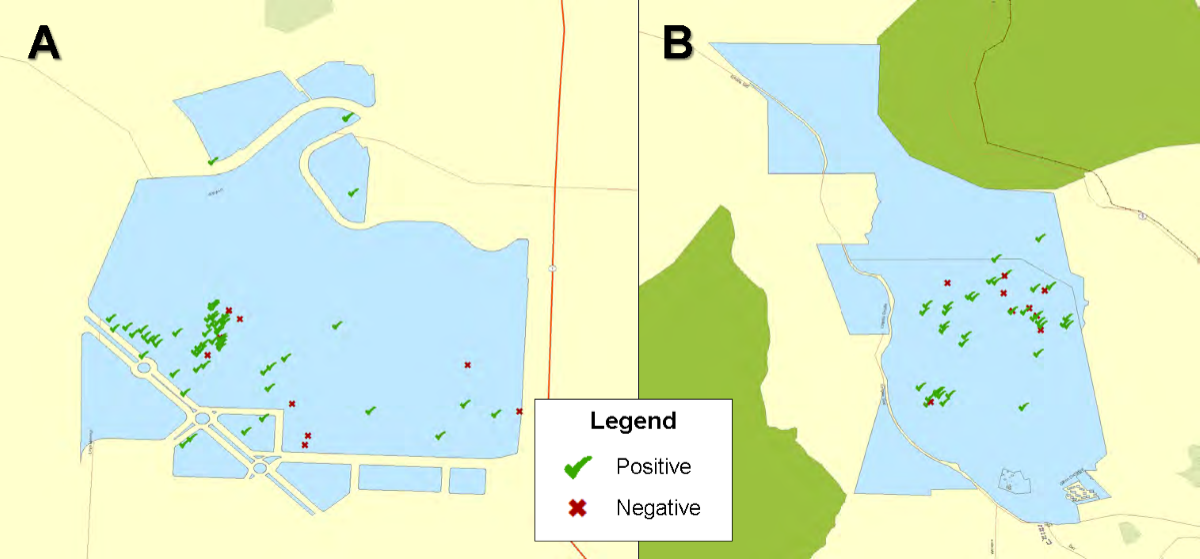
Map depicting geocoding of positive and negative sentiment tweets from California State University (A) and University of California (B) campuses, which had the greatest number of tweets.

A total of 42,877,339 posts with smoking-related keywords were collected from 2015, with 2837 originating from a UC or CSU system campus, and 758 of these manually verified as being associated with user-generated smoking behavior discussions. Among Twitter posts for which positive or negative sentiment was identified (396 posts from 286 unique users), 66.4% (n=89) of posts originating from smoke- and tobacco-free campuses had a positive sentiment toward tobacco products compared to 76.7% (n=201) of posts originating from campuses without smoke- or tobacco-free campus policies (*P*=.03). Figure 1 shows geocoding of positive and negative sentiment tweets from the CSU (A) and UC (B) campuses from which the greatest number of tweets originated.

Four thematic categories of negative sentiment tweets and 9 thematic categories of positive sentiment tweets emerged from the data (Table 1). Content analysis identified 2 dominant themes among negative sentiment tweets on campuses with smoke- and tobacco-free policies: observations of others smoking on campus (likely in violation of existing policies) and displeasure associated with the smell of cigarettes or smokers (Table 2). Assessment of positive sentiment tweets between campuses suggests that the difference in positive sentiment may be driven by the larger number of general comments about one’s own smoking behavior and comments about smoking as a general behavior. A greater variety in types of positive sentiment tweets originating from campuses without a smoke- or tobacco-free policy may have also contributed to differences in sentiment toward smoking between campuses with and those without smoke-free policies; these include urging others to smoke, expression of positive opinions about smoking, and attraction to people who smoke or environments where smoking occurs, though none of these alone were considered dominant themes.

**Table 1:** Thematic categories of smoking-related tweets originating from public 4-year universities by sentiment, California, 2015.

Category		Deidentified examples
**Negative sentiment tweets**		
	Observation of others smoking on campus	*This [person] is smoking a hookah pen in class […] is that necessary* ^a^ *Thanks to all the people who smoke at [deleted]. The second hand smoke is real* ^b^
	Smell of cigarettes or smokers	*Why did [deleted] who smells like cigarettes sit in front of me* ^a^ *[deleted] next to me straight smells like cigarettes & I wanna throw up* ^b^
	Support for smoke- or tobacco-free policy	*It's a no smoking campus [deleted]. #caughingmylungsout* ^a^ *Want to make our campus smoke free hate [deleted]* ^b^
	Opinions against smoking	*Smoking cigarettes is giving [deleted] a death sentence. Guhross* ^b^
**Positive sentiment tweets**		
	Expression of desire to smoke	*tryna smoke but [deleted] is down* ^a^ *Is it too early to smoke?* ^b^
	Report of one’s own smoking	*Smoke break* ^a^ *[That] was [some] strong af hookah* ^b^
	Intention to smoke	*Revising my paper […] as I wait for someone so we can go smoke* ^a^ *If anyone needs me I'll be smoking cigarettes […]* ^b^
	Opposition to campus smoking policy	*[Delete] has a smoke free campus bs man* ^a^
	General comments about one’s smoking behavior	*I don't smoke anymore [delete] ... But i also don't smoke any less* ^b^
	Comments about smoking as general behavior	*Smoke […] eat […] live […]* ^b^
	Urging others to smoke	*@--------------- go smoke with him* ^b^
	Positive opinions about smoking	*[To] all the people smoking cigarettes at least you aren't vaping* ^b^
	Attraction to people who smoke or environments where smoking occurs	*Oddly attracted to how [delete] look smoking a cigarette* ^b^

^a^Tweet originating from a campus with a smoke- or tobacco-free campus policy.

^b^Tweet originating from a campus without a smoke- or tobacco-free campus policy.

**Table 2:** Dominant smoking-related themes originating from public 4-year universities by sentiment and campus smoking policy type, California, 2015.

Campus type	Negative sentiment	Positive sentiment
Campuses with smoke- or tobacco-free policies	Observations of others smoking on campus (n=11) Smell of cigarettes or smokers (n=8)	Expressions of desire to smoke (n=22) Reports of one’s own smoking (n=10) Intention to smoke (n=8)
Campuses without smoke- or tobacco-free policies	Opinions opposed to smoking (n=10) Observations of others smoking on campus (n=8) Smell of cigarettes or smokers (n=7)	Expressions of desire to smoke (n=36) Reports of one’s own smoking (n=23) General comments about one’s own smoking behavior (n=18) Comments about smoking as a general behavior (n=18) Intention to smoke (n=14)

## Discussion

### Principal Findings

Quantitative analysis of tweets originating from campuses of public 4-year universities in California revealed a significant difference in sentiment toward smoking by campus smoking policy status, with a higher proportion of positive sentiment tweets originating from campuses without smoke- or tobacco-free policies, but an overall high percentage of positive sentiment regardless of campus smoking policy type. Greater positive sentiment may have been driven by more tweets containing general comments about one’s own smoking behavior and comments about smoking as a general behavior and a greater variety in types of positive sentiment tweets.

### Comparison With Prior Work

Negative sentiment toward smoking was identified on both campus types, but universities without a smoke- or tobacco-free policy had much higher numbers of positive sentiment tweets in two categories: general comments about one’s own smoking behavior and comments about smoking as a general behavior. These differences may indicate policy passage and implementation at smoke- and tobacco-free campuses may be associated with less positive sentiment toward smoking, consistent with other studies [6,7]. Though efforts such as outreach programs and infrastructure changes may help with policy compliance [23-25], continued effort is needed to change norms around campus smoking and increase policy buy-in. These efforts should be ongoing and consistent, facilitating broad involvement of campus constituents in the policy implementation process. Additionally, twitter posts expressing a desire and intention to smoke as well as self-reported smoking suggest a need to more actively promote cessation on all campuses [24-25]. Future studies should also assess whether sentiment may significantly differ on the basis of different tobacco, e-cigarette, and other smoking product use, particularly in the context of introduction of new products and bans on products (eg, flavored products) that are popular among youth and young adults.

### Limitations

Data collected for this study were limited to Twitter users who enabled geolocation, which may have introduced bias in the volume and types of tweets or Twitter users for whom data were collected. Additionally, this study only reviewed data from a single calendar year to assess differences in sentiment between California universities with and those without smoke- or tobacco-free campus policies, though examining tweets from subsequent years (2016 and 2017) may have yielded additional user sentiment after policy implementation. Another limitation is the constrained contextual information available in some tweets (eg, such as references to “smoking” without mention of a specific product), found mainly among tweets expressing an intention to smoke, which may have referred to marijuana and not tobacco products. We also did not restrict our data set to one tweet per user, though a user expressing negative or positive sentiment may be more likely to have a similar sentiment in other tweets they post. The rationale for this approach included the detection of both second- and first-hand accounts of smoking behavior, which could relate to multiple smoking behavior instances and the possibility that this could lead to possible underestimation of expressed sentiment toward alleged violations of campus smoking bans. Finally, this is an ecological study assessing the relationship between a survey of smoking-related social media posts from college campuses and the policies on those campuses. Associations uncovered in this study may not apply to individuals, and the effect of college policies on sentiment should be further investigated in studies with a breadth of individual participants whose selection is representative of the campus population.

### Conclusions

Among Twitter users in California public universities, the overall sentiment toward tobacco products and use is high, although positive sentiment is higher on campuses without smoke- or tobacco-free campus policies. Smoke- and tobacco-free policy initiatives, including implementation, may help reduce positive sentiment toward smoking on college campuses and should be strengthened to maximally increase their impact.

## Abbreviations

CSU: California State University
UC: University of California
